# Patterns and Drivers of Bumblebee Diversity in Gansu

**DOI:** 10.3390/insects15070552

**Published:** 2024-07-21

**Authors:** Muhammad Naeem, Huanhuan Chen, Wenbo Li, Alice C. Hughes, Paul H. Williams, Nawaz Haider Bashir, Zhengying Miao, Jiaxing Huang, Jiandong An

**Affiliations:** 1 College of Biological Resource and Food Engineering, Qujing Normal University, Qujing 655011, China; 2 State Key Laboratory of Resource Insects, Key Laboratory of Insect-Pollinator Biology of Ministry of Agriculture and Rural Affairs, Institute of Apicultural Research, Chinese Academy of Agricultural Sciences, Beijing 100193, China; 3 Key Laboratory of Yunnan Provincial Department of Education of the Deep-Time Evolution on Biodiversity from the Origin of the Pearl River, Qujing Normal University, Qujing 655011, China; 4 School of Biological Sciences, University of Hong Kong, Hong Kong SAR 999077, China; 5 The Natural History Museum, London SW75BD, UK; 6 Gansu Institute of Apiculture, Tianshui 741022, China

**Keywords:** biodiversity, bumblebees, Gansu, biogeographic regionalization, canonical correspondence analysis

## Abstract

**Simple Summary:**

Bumblebee species are crucial pollinators for both crops and natural ecosystems. Managing diversity and securing the services they provide requires sufficient data to manage these systems effectively. In this study, seventeen environmental factors were selected to understand their contribution to shaping species assemblages and to provide baseline information for understanding drivers of community assembly. The study focused on Gansu Province because it has some of the highest richness of bumblebees within China, and China represents a major data gap for bee distribution data. Net primary productivity followed by water vapor pressure were top-ranked drivers of species distributions. The results enhance our understanding of the importance of environmental factors in shaping bumblebee community assemblages.

**Abstract:**

Understanding the influence of factors responsible for shaping community assemblage is crucial for biodiversity management and conservation. Gansu is one of the richest regions for bumblebee species in the world. We explored the distribution data of 52 bumblebee species collected in Gansu and its surroundings between 2002 and 2022, predicting habitat suitability based on 17 environmental variables using MaxEnt. The factors influencing community assemblage were assessed using canonical correspondence analysis. Net primary productivity, water vapor pressure, temperature seasonality, annual precipitation, and precipitation seasonality were some of the most influential drivers of species distributions. Based on Ward’s agglomerative cluster analysis, four biogeographic zones are described: the Southern humid zone, the Western Qilian snow mountain zone, the Eastern Loess plateau zone, and the Western dry mountain zone. In the clusters of grid cells based on beta diversity values, the Southern humid zone comprised 42.5% of the grid cells, followed by the Eastern Loess plateau zone (32.5%), the Western dry mountain zone (20%), and the Western Qilian snow mountain zone (5%). Almost all the environmental factors showed a significant contribution to the assemblages of bumblebees of different groups. Our findings highlight the need for better data to understand species biogeography and diversity patterns, and they provide key baseline data for refining conservation strategies.

## 1. Introduction

Species assemblages may be shaped by a combination of biogeographic, climatic, and biotic factors, but the relative influence of each is likely to vary considerably [1,2,3,4,5,6]. Climatic factors are known to shape the community assemblage of dung beetles, but few studies exist for other invertebrate taxa [7,8]. Invertebrates provide important services, yet the drivers of their distribution require further research. Bumblebees are key wild pollinators, yet various species are showing population declines [9,10]. Bumblebee community assemblages are impacted by landcover, yet the effect of other factors is still little known [11,12,13].

Gansu Province provides an ideal place to explore drivers of distribution patterns in bumblebees, as 45% of the 125 species recorded in China have been recorded there, one of the highest provincial counts globally [14,15]. For example, the Qinghai-Tibetan plateau has 57 species, whereas South America has 44, the USA has 49, and Canada has 40 bumblebee species [16,17,18,19]. Thus, these data fill a vital gap as China is a hotspot of bee diversity [20], yet, the majority of prior research in Gansu has been limited to basic lists of specimens and species of bumblebees, and higher-resolution analysis is clearly needed [21]. Before 2011, reports on bumblebees of Gansu were reliant on small collections from a few specific parts of the province [22,23,24,25]. Later, a comprehensive report was compiled based on systematic field surveys of bumblebee species collections, which resulted in an increased number of records for Gansu province [26].

Here, we explore bumblebee faunal composition based on bioregionalization at species level across Gansu [25,27]. We analyze (1) patterns of richness of bumblebees in Gansu and (2) map biogeographic regions of bumblebees in Gansu (3) to estimate the contribution of different environmental factors in the assembly of bumblebee communities within each biogeographic zone.

## 2. Materials and Methods

### 2.1. Study Site and Bumblebee Species

**Figure 1:**
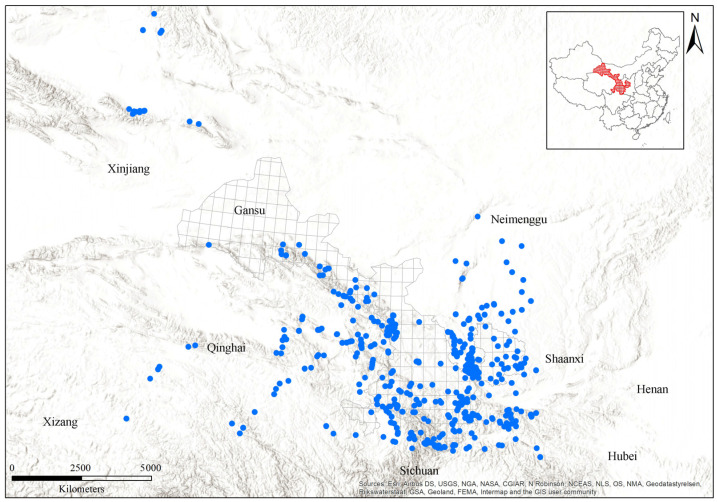
A map of Gansu, with the distribution of bumblebee sampling sites within Gansu and its surroundings. Blue dots represent the collection sites of bumblebees.

Gansu is situated at the northeastern edge of the Qinghai-Tibetan plateau (Figure 1), in a transition zone between the Neimenggu plateau, the Qinghai-Tibetan plateau, and the Loess plateau in China. No bumblebee records were found in northwestern Gansu, so we expanded our study area to include surrounding regions in order to obtain bumblebee records from northwestern regions, increasing the number of species included from 57 within Gansu to 62 (Figure 1). 

More than 8000 specimens of 62 total bumblebee species were recorded from 2295 accessible sampling sites within Gansu and its surrounding regions (Figure 1 and Figure S1 and Table 1). The bumblebee species data used in this study are based on the collection of Institute of Apicultural Research, Chinese Academy of Agricultural Sciences (IAR-CAAS), obtained across Gansu between 2002 and 2022. For the collection of bumblebee species, a range of different environmental conditions was surveyed, including most protected areas and nature reserves across Gansu, to ensure high-quality habitats were included. Collection was conducted from June to September each year, with each site visited by 3-4 non-specialists for 1–2 h on sunny days to maximize bumblebee activity. All specimens were pinned and labeled with individual identifier numbers. Specimens were identified using both a morphological key [24] and COI barcode sequences. All collected specimens were stored in entomological boxes and deposited in the collection of the Institute of Apicultural Research, Chinese Academy of Agricultural Sciences, Beijing, China. The biogeographic zones assessment was based on habitat suitability ranges of modeled bumblebee species within Gansu.

### 2.2. Statistical Analysis

**Figure 2:**
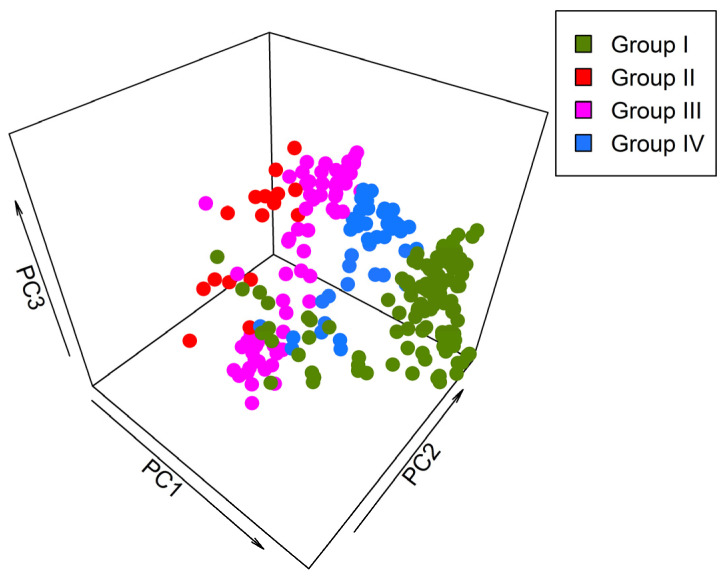
The principal component analysis of 240 grid cells based on 17 environmental variables for bumblebee distribution and assemblage represents the importance of the contributions of all variables in the development of environmental heterogeneity.

The distribution ranges of bumblebee species calculated the pixels of 0.5° longitude × 0.5° latitude within the boundary of Gansu, and there was total of 240 grid cells throughout Gansu (Figure S1) [28]. The values of habitat suitability per grid cell were assessed for all 240 grid cells, and a species vs. sites (grid cells) matrix was developed, where rows represent the grid cells or sites and columns represent the species. Principal component analysis was performed on all 17 variables to better cluster and assess distributional patterns (Figure 2).

For the community assemblage of bumblebee species, Simpson’s dissimilarity was determined for 240 grid cells based on predicted habitat suitability values per grid cell within the Gansu of each of the bumblebee species using the “vegan” and “betapart” packages in R version 4.3.3 [29,30]. This calculation was made based on the following formula:

$$\beta sim=\frac{\min\left(b,\;c\right)}{a+\min\left(b,\;c\right)}$$

**Figure 3:**
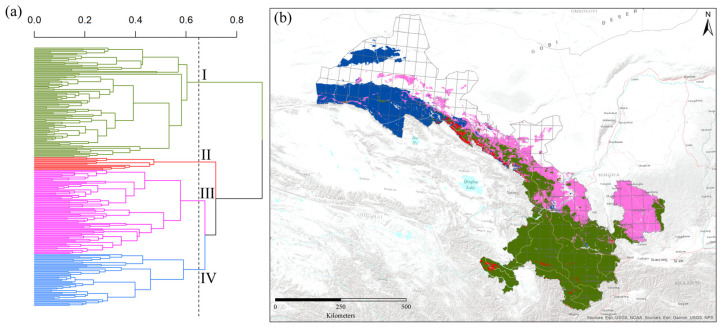
The classification of 240 grid cells dominated by different bumblebee species into four groups (zones) using Ward’s agglomerative clustering analysis, and phenon lines represent the biogeographic groups. Here, I represents the Southern humid zone, II represents the Western Qilian snow mountain zone, III represents the Eastern Loess plateau zone, and IV represents the Western dry mountain zone (**a**), and the hotspot regions of each group’s bumblebee species are shown with colorful grid cells in Gansu of China (**b**).

**Figure 4:**
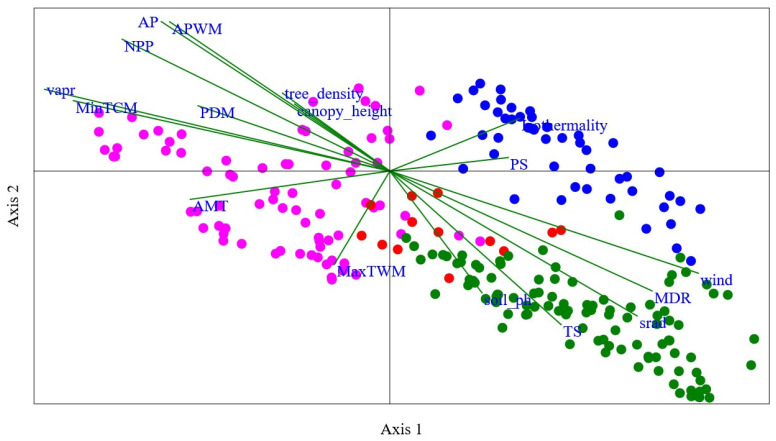
Canonical Correspondence Analysis (CCA) plot illustrating the relationship between bumblebee species composition and environmental variables within Gansu. Each point represents a sampling site within Gansu, and their positions are determined by the scores on axis 1 and axis 2. The length and direction of lines depict the importance and direction of each variable’s influence on the species composition. Here, “NPP” represents the Net primary productivity, “srad” represents the solar radiation, “vapr” represents the vapor pressure, “AMP” represents annual mean temperature, “MDR” represents mean diurnal range, “TS” represents temperature seasonality, “MaxTWM” represents maximum temperature of wettest month, “MinTCM” represents minimum temperature of coldest month, “AP” represents annual precipitation, “APWM” represents annual precipitation of wettest month, and “PDM” represents precipitation of driest month. Here, green, red, pink, and blue dots represent the Southern humid zone, the Western Qilian snow mountain zone, the Eastern Loess plateau zone, and the Western dry mountain zone, respectively.

Here, the number of species shared by the two grid cells is represented by “*a*”, the unique number of species to one grid cell is represented by “*b*”, and the unique number of species to another grid cell is represented by “*c*”. Based on the dissimilarity values, Ward’s agglomerative clustering analysis was used to classify the grid cells into different groups [31] (Figure 3a,b). To explore the impact of environmental heterogeneity, we used Canonical Correspondence Analysis (CCA) in the “vegan” package of R 3.3.3 [29,30] (Figure 4). CCA was used here for the analysis of environmental heterogeneity because it can assess that multiple variables are influenced simultaneously on the species assemblage. The permutation test is used to assess the statistical significance of the CCA axes. Furthermore, the strength and direction of the relationship between environmental factors and community assemblage can be calculated [32,33].

Whilst our study provides an exploration of distribution and zones of bumblebees in Gansu, it was not without limitations. Given persistent data gaps [34], modeling can provide a key mechanism to overcome spatial sampling gaps, when used with caution. The sampling of bumblebees is difficult in most of Gansu because in more arid environments species are patchily distributed and much more difficult to collect [19,20,21].

### 2.3. Model Development and Assessment

The suitability ranges were assessed using MaxEnt software *v.* 3.4.4 [35]. MaxEnt is widely used to assess species distribution. It works on the major assumption of presence-only data, meaning it does not require absence data, and requires environmental variables that are most relevant to the species [35]. To model the distribution of bumblebee species, we selected environmental variables that are most likely to drive species distributions and their reproductive success of species, and these are cross-referenced with the distribution of each species to assess the habitat requirements of each species and then to assess where each species may occur [19,20] (see Table S1 for variables and sources). 

For autocorrelation, SDMtoolbox in ArcMap *v*. 10.3 was used to calculate Pearson’s correlation coefficients (r < 0.9) [36], and a set of 17 variables was selected. These included bioclimatic factors, tree density, canopy height, soil pH, water vapor, and solar radiation factors, using similar factors as previous studies [37]. We did not include elevation because it is used to downscale bioclimatic factors [38]. The model evaluation was assessed on the basis of the area under the curve (AUC) of the receiver operating characteristic (ROC) curves [39]. 

## 3. Results

### 3.1. The Contribution of Environmental Factors in the Habitat Suitability of Bumblebee Species

**Figure 5:**
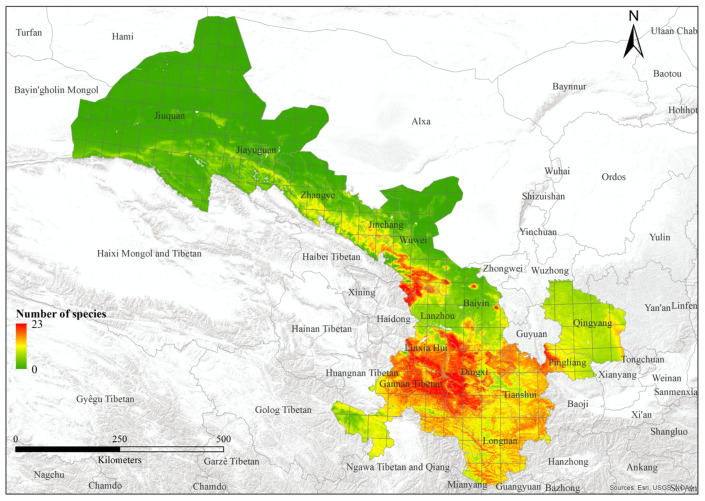
Species richness of bumblebees within Gansu of China.

We modeled 52 out of 62 bumblebee species, excluding 10 species with less than three records of distribution. Species varied from 0 to 23 (Figure 5; Table 1). The area under the curve values of training data vary from 0.94 to 0.99, whereas the range of values of test data varies from 0.526 to 1. The 10th-percentile training presence threshold values showed that the predicted suitable areas vary from 0.003 to 0.188 (Table S2). However, the factor with the highest contribution varies for each species. For example, for 87% of bumblebees, the most important factor was “Net primary productivity”, and in 65% of species, this factor showed top contribution in the distribution of species. Water vapor pressure was the top contributing factor for 10% of bumblebee species. Additionally, “Vegetation height”, “Solar radiation”, and “Tree density” contributed 2% each in the distribution of bumblebee species. Various bioclimatic factors were also important; “TS” (temperature seasonality), “AP” (annual precipitation), and “PS” (precipitation seasonality) accounted for 5% each in the distribution of species. Similarly, “MaxTWM” (maximum temperature of warmest month) exhibited a 4% contribution to the distribution of bumblebee species, whereas “MinTCM” (minimum temperature of coldest month) and “PWM” (precipitation of wettest month) contributed 2% each to the overall species distribution pattern. Further details are present in the Supplementary Materials (Table S2).

Using principal component analysis (PCA), the first three axes included 79.24% of total variation (Figure 2). PC1 gave a substantial proportion variation, almost 48.41% among 240 grid cells of Gansu. PC2 accounts for 22.82% environmental heterogeneity, and PC3 accounts for 8.01% environmental heterogeneity. Distinct clustering patterns emerged in 3D scatter plot representations of PCA (Figure 2). The spatial distribution of the grid cells of different groups in scatter plot representations show similar kinds of clustering, as displayed in CCA analysis (Figure 4). 

### 3.2. Biogeographic Zones of Bumblebee Community Assemblage

Based on predicted values of habitat suitability modeling, Ward’s agglomerative cluster analysis segregated the species into four distinct groups (Figure 3a). In the clusters of grid cells based on beta diversity values, the Southern humid zone (green) comprised 42.5% of the grid cells, followed by the Eastern Loess plateau zone (pink) (32.5%), the Western dry mountain zone (blue) (20%) and the Western Qilian snow mountain zone (red) (5% of the grid cells). A phenon line was also drawn on the clusters to visualize the four major bumblebee biogeographic regions based on the cophenetic correlation coefficient value (r = 0.65) (Figure 3a). The relative positions of the four bumblebee biogeographic regions of bumblebees in Gansu are displayed in Figure 3b. The bumblebee species found only in the Southern humid zone are *Bombus atripes*, *B. bicoloratus*, *B. trifasciatus*, *B. flavescens*, *B. breviceps*, *B. hengduanensis*, *B. remotus*, *B. grahami*, *B. festivus*, etc., which is the relative moist group of South China. *B. lantschouensis*, *B. ganjsuensis*, *B. ignitus*, *B. pyrosoma*, *B. melanurus*, *B. sibiricus*, *B. opulentus*, *B. longipes*, *B. hedini*, etc., are mainly distributed in the Eastern Loess plateau zone, which belongs to the temperate group of North China. The species limited to the Western Qilian snow mountain zone are *B. validus*, *B. wangae*, *B. waltoni*, *B. tibetanus, B. prshewalskyi*, *B. supremus*, *B. personatus*, *B. qilianensis*, *B. kashmirensis*, *B. minshanicola*, and *B. convexus*, most of which are Qinghai-Tibetan plateau species with high elevation. Compared to the above three groups, few species, such as *B. asiaticus* and *B. difficillimus*, are restricted to the Western dry mountain zone of the Central Asian group.

### 3.3. Bumblebee Community Assemblage Explained by Environmental Heterogeneity

In our set of 17 environmental variables (please see Table S1), simple linear regression analysis indicates that bee presence in the Southern humid zone is significantly (*p* < 0.05) positively related to NPP, isothermality, AP, PWM, PDM, and PS. In the Eastern Loess plateau zone, the presence of bees is significantly related to NPP, AP, PWM, PDM, MinTCM, tree density, and AMT. In the Western dry mountain zone, the presence of bumblebee species is positively and significantly related to NPP, AP, PWM, MinTCM, AMT, and vapr. Finally, the presence of species in the Western Qilian snow mountain zone is positively associated with NPP, AP, and PWM.

There are no bumblebees found in the northwest part of Gansu. The main factor is that this area is a very dry desert. The relationship between bumblebee community assemblage and environmental variables is also shown in Figure 4 based on Canonical Correspondence Analysis (CCA) constrained space. The eigenvalues indicate that axis 1, representing 38.35% of the variance, plays a substantial role in explaining the observed patterns of community assemblage. Axis 2, with 18.83% of the variance, contributes further to the understanding of species–environment relationships. Together, these two axes explain a significant portion (57.18%) of the total variance present between the species assemblage of different sampling sites. In our results, the permutation “*p*” value for both axis 1 and axis 2 is 0.001. This low *p*-value suggests that the observed relationship between bumblebee species assemblage and environmental variables on these axes is statistically significant (Figure 4). 

The most important variables found to be highly related in the regionalization of the Southern humid zone and the Western Qilian snow mountain zone are soil pH, temperature seasonality, and solar radiation. Regarding the regionalization of the Eastern Loess plateau zone, the most important variables are average mean temperature (AMT), tree density, maximum temperature of warmest month (MaxTWM), and Net primary productivity (NPP). Similarly, for the region of Western dry mountain, the most important variables found were precipitation seasonality (PS) and isothermality. Axis 1 of the CCA plot showed a strong positive relationship between the species and environmental factors such as wind (with the values of 0.87), mean diurnal range (0.74), solar radiation (0.70), temperature seasonality (0.48), isothermality (0.36), precipitation seasonality (0.33), and soil pH (0.26). However, the variables average precipitation (0.86), average precipitation of wettest month (0.85), Net primary productivity (0.76), water vapor pressure (0.47), tree density (0.45), minimum temperature of coldest month (0.40), canopy height (0.38), precipitation of driest month (0.37), isothermality (0.30), and precipitation seasonality (0.08) showed a strong positive relationship on axis 2 of the CCA plot (Figure 4).

## 4. Discussion

Studying the patterns and drivers of assemblage of bumblebee species on a local and fine scale is crucial in enabling local-scale management [20]. Climate is the main factor driving species distributions and community assembly in bees in Gansu (Table S2 and Figure 4) [40,41]. Within our analysis, all bumblebee species showed major responses to climate variables, except for *Bombus grahami*, though this may be due to undersampling, as this area represents the northern limit of its range [41]. 

The variable importance varied between species, for example, the most influential factor, Net primary productivity (NPP), was found to be influential in 87% of bumblebee species and the top driver for 65% of bumblebee species. However, NPP was not an important factor for *B. atripes*, *B. difficillimus*, *B. festivus*, *B. semenovi*, *B. supremus*, *B. tanguticus*, and *B. tibetanus*. Some of these species, such as *B. atripes* (a rare species in South China) and *B. festivus*, are southern species that inhabit humid regions with forests. Thus, the data here may be insufficient for analysis, as we are only exploring the distribution on the edge of their range [40]. However, whilst NPP is important, relationships are nuanced, especially in areas without significant tree-cover such as Gansu (and non-forest NPP is known to be a more significant driver than NPP overall) [20]. 

Solar radiation was selected as a variable for 72% of bumblebee species, possibly due to its influence on plant growth and bee thermoregulation [42]. Wind speed also contributes to the distribution of 77% of bumblebee species, and water vapor pressure contributes to the distribution of 79% of bumblebee species and may impact flight control of bumblebee species [43]. Tree canopy height was also incorporated in our study, because dense wooded systems have different floral provisioning and structures than open habitats [44]. 

The highest species richness was observed in Lintan County, in the transition zone between the western Qinghai-Tibetan Plateau and the Eastern Loess plateau. This area is characterized by varied topography and diverse habitats, encompassing extensive natural environments with mountains and grasslands to agricultural areas. This combination likely contributes to a high turnover in species presence. Areas with the highest species richness (>20) come under the climatic zone of temperate monsoon, and highly seasonal habitats can often support high diversity [45]. Further, it has been found that temperate areas support higher bumblebee richness than tropical areas [14]. Gansu is a temperate region. But part of southernmost Gansu is sub-tropical. 

In our study, the Gansu was divided into four bumblebee biogeographic zones (Figure 3b). Similar studies have divided the whole of China into four bumblebee biogeographic regions, though we increased resolution to explore distribution on more regional scales [46]. Within China, the four biogeographic zones of Gansu fall under the North China and Tibetan Plateau regions of bumblebee groups. At a larger scale, the world was divided into 10 principal biogeographic regions of bumblebee species [1], and our four zones of Gansu fall under the category of the northern Oriental region. Another study categorized the bumblebee species of the Tibetan plateau into three principal groups, a distinct group of Himalayan faunas in the south, an interior Tibetan fauna, and the Qinghai and Gansu fauna of the northeast [19]. The Himalayan faunas and Qinghai and Gansu faunas showed closer association with climatic factors, whereas the interior Tibetan fauna showed weaker association [19].

Sampling bumblebees in Gansu is challenging due to the region’s complex topography and arid environment in the northwest, which limited sampling in some areas. In arid areas, species tend to be patchily distributed, making collection efforts more difficult [19]. Consequently, certain grid cells of dry mountains and deserts lacked bumblebee samples, and further research is needed for a more comprehensive understanding of regional distributions (Figure 1).

This study explains the most basic elements of distributional dynamics and the assemblage of bumblebee species at a regional scale, which are unknown for many insects [47]. The information about the faunal composition and contribution of different factors enables us to consider those factors while designing conservation strategies and management decisions.

## Figures and Tables

**Table 1:** Bumblebee species of different subgenera collected from Gansu and its surrounding areas. The species with “*” were excluded in the habitat suitability modeling because of their records < 3.

Sr. No	Species	Subgenus
1	*B. asiaticus*	*Sibiricobombus*
2	*B. atripes*	*Thoracobombus*
3	*B. bellardii*	*Psithyrus*
4	*B. bicoloratus*	*Megabombus*
5	*B. bohemicus*	*Psithyrus*
6	*B. breviceps*	*Alpigenobombus*
7	*B. campestris*	*Psithyrus*
8	*B. chinensis*	*Psithyrus*
9	*B. consobrinus*	*Megabombus*
10	*B. convexus*	*Mendacibombus*
11	*B. coreanus* *	*Psithyrus*
12	*B. cornutus*	*Psithyrus*
13	*B. czerskii* *	*Megabombus*
14	*B. deuteronymus*	*Thoracobombus*
15	*B. difficillimus*	*Subterraneobombus*
16	*B. expolitus* *	*Psithyrus*
17	*B. festivus*	*Melanobombus*
18	*B. filchnerae*	*Thoracobombus*
19	*B. flavescens*	*Pyrobombus*
20	*B. grahami*	*Alpigenobombus*
21	*B. hedini*	*Thoracobombus*
22	*B. humilis*	*Thoracobombus*
23	*B. hengduanensis*	*Pyrobombus*
24	*B. ignitus*	*Bombus*
25	*B. imitator* *	*Thoracobombus*
26	*B. impetuosus*	*Thoracobombus*
27	*B. infrequens* *	*Pyrobombus*
28	*B. kashmirensis*	*Alpigenobombus*
29	*B. qilianensis*	*Melanobombus*
30	*B. koreanus*	*Megabombus*
31	*B. ladakhensis* *	*Melanobombus*
32	*B. laesus*	*Thoracobombus*
33	*B. lantschouensis*	*Bombus*
34	*B. lemniscatus*	*Pyrobombus*
35	*B. lepidus*	*Pyrobombus*
36	*B. minshanicola*	*Bombus*
37	*B. longipes*	*Melanobombus*
38	*B. melanurus*	*Subterraneobombus*
39	*B. minshanensis*	*Bombus*
40	*B. norvegicus* *	*Psithyrus*
41	*B. opulentus*	*Thoracobombus*
42	*B. ganjsuensis*	*Bombus*
43	*B. personatus*	*Subterraneobombus*
44	*B. picipes*	*Pyrobombus*
45	*B. pyrosoma*	*Melanobombus*
46	*B. religious* *	*Megabombus*
47	*B. remotus*	*Thoracobombus*
48	*B. prshewalskyi*	*Melanobombus*
49	*B. rupestris* *	*Psithyrus*
50	*B. semenovi*	*Sibiricobombus*
51	*B. sibiricus*	*Sibiricobombus*
52	*B. sichelii*	*Melanobombus*
53	*B. skorikovi*	*Psithyrus*
54	*B. supremus*	*Megabombus*
55	*B. sushkini*	*Megabombus*
56	*B. tanguticus*	*Melanobombus*
57	*B. tibetanus*	*Psithyrus*
58	*B. trifasciatus*	*Megabombus*
59	*B. turneri* *	*Psithyrus*
60	*B. validus*	*Alpigenobombus*
61	*B. waltoni*	*Mendacibombus*
62	*B. wangae*	*Pyrobombus*

## Data Availability

All the data generated or analyzed during this study are included in this published article and its Supplementary Materials.

## Supplementary Materials

The following supporting information can be downloaded at: https://www.mdpi.com/article/10.3390/insects15070552/s1, Figure S1: Habitat suitability modeling of bumblebee species of Gansu. X-axis represent the longitude and Y-axis are latitude. Here the values closer to 1 showed more suitability range of a species; Table S1: The environmental variables utilized in the investigation of the bumblebee biotic com-munity assemblage. The spatial resolution is approximately 1 km^2^; Table S2: Area under the curve values at training and test data, fractional predicted area and the highest contribution of environmental factors in the spatial distribution modeling of modelled bumblebee species of Gansu and its surrounding areas. Here, ‘PS’ stands for precipitation seasonality, ‘NPP’, Net primary productivity, ‘vapr’, vapor pressure, ‘TS’, temperature seasonality, ‘MaxTWM’, maximum temperature of warmest month, ‘AP’ average precipitation, ‘PWM’ precipitation of wettest month. Refs. [39,48] are mentioned in Supplementary Materials.
