# Supplementary material for: Patterns and Drivers of Bumblebee Diversity in Gansu
Source: Insects. 2024 Jul 21;15(7):552. doi: 10.3390/insects15070552 (PMC11276862; doi:10.3390/insects15070552)
Supplement: Supplementary file 1 [file insects-15-00552-s001.zip › insects-3035416-supplementary.pdf]

Supplementary Materials

Table S1. The environmental variables utilized in the investigation of the bumblebee biotic community assemblage. The spatial resolution is approximately 1 km^2.

Sr. No.	Environmental Variable	Acronym	Units	Source
1	Annual Mean Temperature	AMT	**°C**	www.worldclim.org
2	Mean Diurnal Range (Mean of Monthly (max temp – min temp))	MDR	**°C**	www.worldclim.org
3	Isothermality (BIO2/BIO7) (* 100)	Isothermality	**°C**	www.worldclim.org
4	Temperature Seasonality (standard deviation *100)	TS	**°C**	www.worldclim.org
5	Max Temperature of Warmest Month	MaxTWM	**°C**	www.worldclim.org
6	Min Temperature of Coldest Month	MinTCM	**°C**	www.worldclim.org
7	Annual Precipitation	AP	mm	www.worldclim.org
8	Precipitation of Wettest Month	PWM	mm	www.worldclim.org
9	Precipitation of Driest Month	PDM	mm	www.worldclim.org
10	Precipitation Seasonality (Coefficient of Variation)	PS	mm	www.worldclim.org
11	Solar Radiation (kJ m-2 day-1)	srad	$kJm^{-2}day^{-1}$	www.worldclim.org
12	Wind Speed (m s-1)	wind	ms^{-1}	www.worldclim.org
13	Water Vapor Pressure (kPa)	vapr	kPa	www.worldclim.org
14	Net Primary Productivity	NPP	g C/m^2/year	[1]
15	Tree Density		trees/km^2	[2]
16	Vegetation Height	Canopy height		https://landscape.jpl.nasa.gov/data/
17	Soil pH			https://files.isric.org/soilgrids/latest/data_aggregated/1000m/

Table S2. Area under the curve values at training and test data, fractional predicted area and the highest contribution of environmental factors in the spatial distribution modeling of modelled bumblebee species of Gansu and its surrounding areas. Here, 'PS' stands for precipitation seasonality, 'NPP', Net primary productivity, 'vapr', vapor pressure, 'TS', temperature seasonality, 'MaxTWM', maximum temperature of warmest month, 'AP' average precipitation, 'PWM' precipitation of wettest month.

Sr. No.	Species Name	AUC of Training data	AUC of Test data	Fractional predicted area at 10th percentile training presence	Highest contributing factor in the modeling (%)
1	*B. asiaticus*	0.984	0.918	0.046	NPP (40%)
2	*B. atripes*	0.99	0.992	0.020	vapr (73.8%)
3	*B. bellardii*	0.99	0.99	0.004	NPP (35.2%)
4	*B. bicoloratus*	0.992	0.991	0.022	NPP (48%)
5	*B. bohemicus*	0.964	0.526	0.067	TS (44.9%)
6	*B. breviceps*	0.994	0.997	0.014	vapr (29.9%)
7	*B. campestris*	0.982	0.992	0.036	NPP (49.7%)
8	*B. chinensis*	0.995	0.980	0.015	NPP (58.4%)
9	*B. consobrinus*	0.989	0.989	0.031	NPP (54.6%)
10	*B. convexus*	0.997	0.990	0.009	NPP (39.5%)
11	*B. cornutus*	0.981	0.985	0.037	NPP (93.5%)
12	*B. deuteronymus*	0.986	0.989	0.029	NPP (52.7%)
13	*B. difficillimus*	0.963	0.887	0.083	MaxTWM (45.6%)
14	*B. festivus*	0.999	0.997	0.003	srad (63.2%)
15	*B. filchnerae*	0.974	0.947	0.075	NPP (59.4%)
16	*B. flavescens*	0.985	0.993	0.188	vapr (31.2%)
17	*B. ganjsuensis*	0.988	0.985	0.035	NPP (41.2%)
18	*B. grahami*	0.992	1	0.025	Canopy height (82%)
19	*B. hedini*	0.983	0.964	0.050	NPP (46.2%)
20	*B. humilis*	0.993	0.973	0.020	NPP (44.9%)
21	*B. hengduanensis*	0.969	0.951	0.107	NPP (55.2%)
22	*B. ignitus*	0.985	0.963	0.040	AP (26.6%)
23	*B. impetuosus*	0.988	0.979	0.036	NPP (44%)
24	*B. kashmirensis*	0.962	0.917	0.115	AP (24.7%)
25	*B. qilianensis*	0.952	0.913	0.130	NPP (52.6%)
26	*B. koreanus*	0.985	0.983	0.022	NPP (61.3%)
27	*B. laesus*	0.993	0.996	0.029	NPP (57.2%)
28	*B. lantschouensis*	0.983	0.964	0.042	NPP (26%)
29	*B. lemniscatus*	0.969	0.911	0.079	TS (48.8%)
30	*B. lepidus*	0.974	0.985	0.073	NPP (52.5%)
31	*B. minshanicola*	0.980	0.982	0.066	NPP (55.2%)
32	*B. longipes*	0.984	0.985	0.043	NPP (48.3%)
33	*B. melanurus*	0.971	0.941	0.083	NPP (38.7%)
34	*B. minshanensis*	0.986	0.969	0.040	NPP (53.9%)
35	*B. opulentus*	0.993	0.963	0.012	vapr (56.7%)
36	*B. personatus*	0.965	0.923	0.120	NPP (50.7%)
37	*B. picipes*	0.986	0.974	0.033	NPP (59.9%)
38	*B. pyrosoma*	0.973	0.967	0.067	MinTCM (24.7%)
39	*B. remotus*	0.992	0.978	0.026	Tree density (48.3%)
40	*B. prshewalskyi*	0.964	0.974	0.078	NPP (53.1%)
41	*B. semenovi*	0.985	0.930	0.023	vapr (53.5%)
42	*B. sibiricus*	0.970	0.960	0.073	NPP (33.6%)

43	*B. sichelii*	0.974	0.975	0.062	NPP (54.5%)
44	*B. skorikovi*	0.968	0.941	0.070	NPP (42.6%)
45	*B. supremus*	0.942	0.895	0.091	AP (38.4%)
46	*B. sushkini*	0.971	0.954	0.050	NPP (39.8%)
47	*B. tanguticus*	0.991	0.856	0.021	MaxTWM (58.8%)
48	*B. tibetanus*	0.973	0.998	0.055	PWM (49.6%)
49	*B. trifasciatus*	0.993	0.990	0.015	NPP (38.4%)
50	*B. validus*	0.990	0.974	0.030	TS (49.2%)
51	*B. waltoni*	0.955	0.902	0.112	NPP (36%)
52	*B. wangae*	0.994	0.967	0.019	NPP (47.5%)

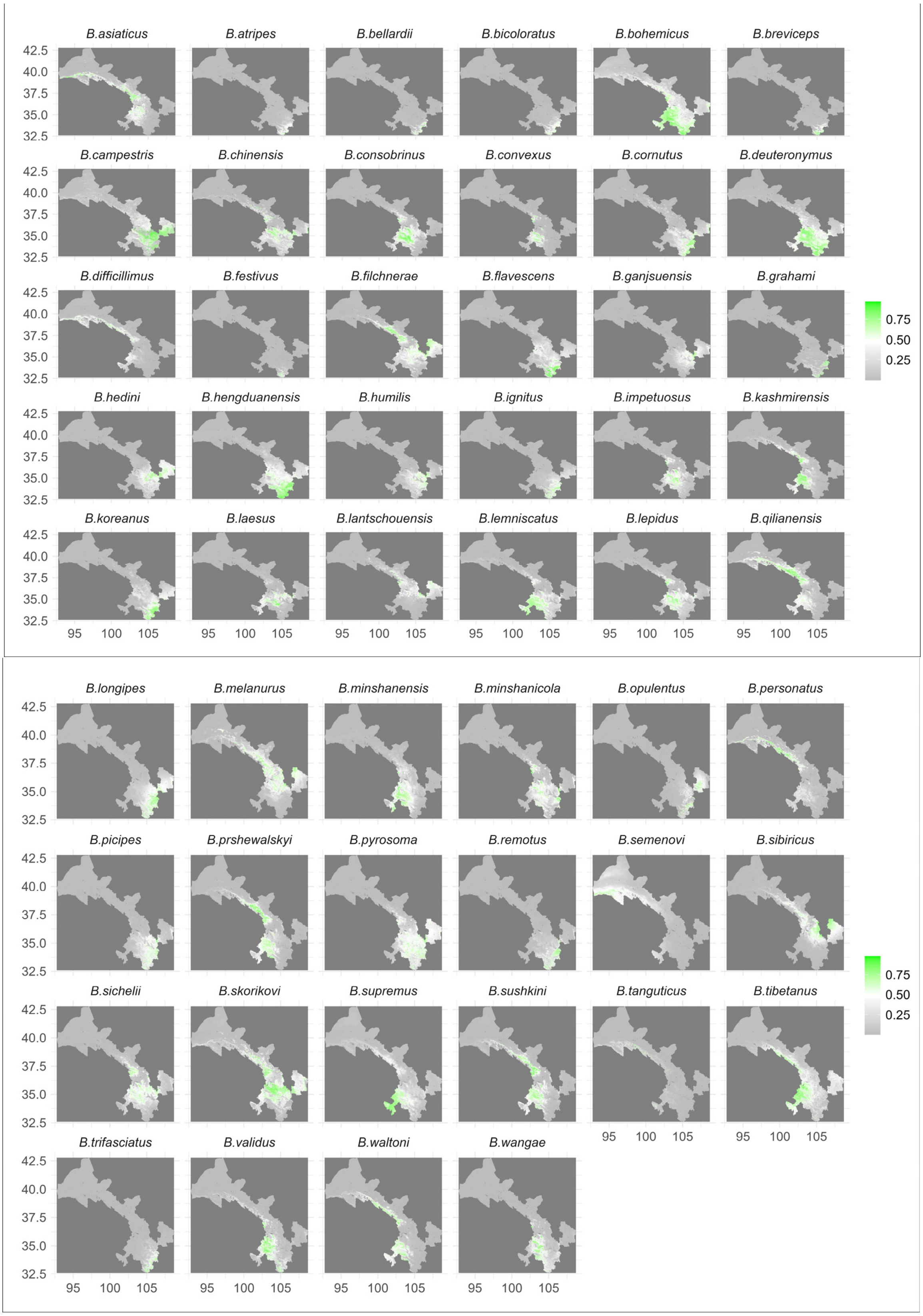

Figure S1. Habitat suitability modeling of bumblebee species of Gansu. X-axis represent the longitude and Y-axis are latitute. Here the values closer to 1 showed more suitablity range of a species.

References

1. FAO (Food and Agriculture Organization of the United Nations). FAO Global Information System on Water and Agriculture (Aquastat)—Evaporation. Available online: https://data.apps.fao.org/catalog/dataset/dce0415f-fd48-468f-a197-e4603dbf88dc/resource/be9143d9-034b-46c9-8b8d-b92f82110dc2?inner_span=True (accessed on 11 April 2024).
2. Crowther, T.W.; Glick, H.B.; Covey, K.R.; Bettigole, C.; Maynard, D.S.; Thomas, S.M.; Smith, J.R.; Hintler, G.; Duguid, M.C.; Amatulli, G.; et al. Global Map of Tree Density; 2016; https://doi.org/10.6084/m9.figshare.3179986.v2 (accessed on 11 April 2024).
